# Burden of disease in Nariño, Colombia, 2010

**Published:** 2014-09-30

**Authors:** Anderson Rocha-Buelvas, Elizabeth Trujillo-Montalvo, Carlos Hidalgo-Patiño, Angela Hidalgo-Eraso

**Affiliations:** 1 Consultant of the Study on Burden of Disease in the Department of Nariño. Pasto. Colombia; 2 Instituto Departamental de Salud de Nariño. Pasto. Colombia

**Keywords:** Cost of disease, health priorities, epidemiology, Colombia, communicable diseases, chronic disease, violence, health planning

## Abstract

**Objective:**

This study sought to measure burden of disease and identifies health priorities from the Disability Adjusted Life Years (DALYs) indicator.

**Methods:**

This is the first study on burden of disease for a department in Colombia by using a standardized methodology. By using the

DALYs indicator, burden of disease was identified in the department of Nariño according to the guidelines established by the World Health Organization.

**Results:**

The DALYs in the Department of Nariño highlight the emergence of communicable, maternal, perinatal, and nutritional diseases during the first years of life; of accidents and lesions among youth, and non-communicable diseases in older individuals. Also, accidents and lesions are highlighted in men and non-communicable diseases in women.

**Conclusions:**

This study is part of the knowledge management process in the Departmental Health Plan for Nariño - Colombia 2012-2015 and contributes to the system of indicators of the 2012 ten-year public health plan. This research evidences that communicable diseases generate the biggest part of the burden of disease in the Department of Nariño, that DALYs due to non-communicable diseases are on the rise, and that accidents and lesions, especially due to violence are an important cause of DALYs in this region, which is higher than that of the country.

## Abstract (es)

**Objetivo:**

Medir la carga de enfermedad e identificar las prioridades de salud a partir del indicador Años de Vida Saludables Perdidos (AVISA).

**Métodos:**

Mediante el uso del indicador AVISA, identificar la carga de enfermedad en el departamento de Nariño según la Guía de la Organización Mundial de la Salud.

**Resultados:**

Este es el primer estudio de carga de enfermedad de un departamento en Colombia que utiliza una metodología estandarizada. Los AVISA en el Departamento de Nariño destacan la emergencia de las enfermedades transmisibles, maternales, perinatales y nutricionales en los primeros años de vida; de los accidentes y lesiones entre los jóvenes y las enfermedades no trasmisibles en individuos mayores; así como los accidentes y lesiones destacan en los hombres y las enfermedades no transmisibles en las mujeres.

**Conclusiones:**

Este estudio es parte del proceso de gestión de conocimiento del Plan de Salud Departamental de Nariño -Colombia 2012-2015 y contribuye al sistema de indicadores del plan decenal de salud pública 2012. Esta investigación evidencia que las enfermedades trasmisibles generan la mayor parte de la carga de enfermedad del Departamento de Nariño, que los AVISA por enfermedades no transmisibles están en ascenso, y que los accidentes y lesiones, especialmente por causa de la violencia son una causa importante de AVISA en esta región, siendo superior a la del país.

## Introduction

It is important to clarify that the epidemiological panorama within the global environment has been transformed in recent decades and, currently, it comprises a broad combination of communicable and non-communicable pathologies along with accidents and lesions, whose complexity in its care requires organizational schemes of healthcare systems to respond to new health challenges 1. Consequently, identifying and monitoring these tendencies of new epidemiological profiles requires methodologies different from the traditional analyses of mortality.

Thus is how the main purpose of a health research system in a country like Colombia and in each of its regions must focus on the analysis of the health situation, preferably as proposed by the World Health Organization (WHO) since the 1990s. Hence, analyses of cost effectiveness, analyses of equity, and studies of burden of disease contribute to the formulation of public policies. This indicator, devised within Harvard's School of Public Health in collaboration with the World Bank and the World Health Organization, was used for the first time in the 1993 World Bank report. Said report permitted identifying health problems that without being causes of death are causes of important morbidity, co-morbidity, and disabilities, given that such integrate the burden produced by premature mortality, duration, and sequelae of disease and disability associated to damages, all through the Disability Adjusted Life Years (DALYs) indicator 2.

Antecedents in Colombia regarding the estimation of the burden of disease date back to 1995 3 and 2005 4; the first was carried out through direct estimations of DALYs for mortality and indirect estimations of DALYs for disability 3, whose reference was the study of burden of disease in Mexico in 1994 5; and the latter was carried out by using a methodology employed by the Center of Projects for Development (CENDEX) 4 in Mexico in 2002 for the Mexican Social Security Institute (IMSS) 6.

Within the current scenario that debates on a possible modification to Legislation 100 of 1993, it is important to update studies on burden of disease existing in the country and its regions and even consolidate a model for its periodic and frequent calculation, complemented with an assessment of the epidemiological profile and the costs of our benefit plans (Obligatory Health Plan of the contributive scheme for individuals who are employed and of the subsidized scheme for individuals with low economic resources). In the Americas, countries like Mexico, Chile, Brazil, Costa Rica, Peru 7, and Colombia 4 have already conducted studies on burden of disease, revealing that the main causes of disease are those from the group of non-communicable diseases 7. Based on the DALYs indicator, globally it has been found that the 10 principal causes of disease are unipolar major depression, alcohol consumption, asthma, dental caries, cardiovascular diseases, and diabetes; all belonging to the group of non-communicable diseases, as well as asphyxia and trauma at birth in the group of communicable diseases, and violence and aggressions in the group of lesions 8. The panorama of these countries is different within them, as shown by this study for the Colombian case.

It is worth mentioning that DALYs, according to the WHO, are input estimators that keep precise mathematical relationships framed within a compartmental model, whose internal coherence can be verified. Said model classifies individuals in a population into four states or basic compartments determined by the health status, disease or death (the last is, in turn, divided into death due to disease under study and death due to other causes). Thus, the population may be contemplated as a set of individuals subdivided into groups with distinct states of health or in distinct stages of vital processes, among which transactions or population movements are established, like: the occurrence of new cases of disease, occurrence of death, births, ageing of individuals, recovery from disease, persistence of the disease status, and establishment of sequelae). Another advantage of the model by the WHO is that it permits identifying mortality attributable to a specific disease by measuring excessive deaths with respect to the population without illness, assuming that mortality due to other causes is equal in the population without illness and in the population with illness. However, one of the limitations of these types of studies - as evidenced in prior studies in Colombia 3 ^,^ 4 and Latin America 9 - is that to obtain the estimators of burden of disease we require information that is subject to the availability and quality of data, bureaucracy, and the technical capacity of the institutions responsible for the sources of information, as we see with the Individual Registry of Service Provision (RIPS, for the term in Spanish), whose access depends on the Information Management System of Colombia's Ministry of Health and Social Protection, which in previous years had greater deficiencies. Besides the deficiencies of the information systems, another source of limitations to establish the burden of disease refers to the need to achieve a precise evaluation 10 ^,^ 11, which is carefully reviewed in the study methodology.

## Materials and Methods

A population-type descriptive study was conducted, following the methodology from the document "National burden of disease studies: a practical guide" by the World Health Organization (WHO) in 2001, using an indicator proposed by Murray and López in 1996 denominated DALYs (potentially lost life years) 12. The study used two population databases, Individual Registry of Service Provision (RIPS) y el record of vital statistics (VS).

To obtain the burden of disease estimators, certain information input was required, like specific mortality due to causes for each disease whose burden was to be assessed, accumulated incidence of each of these diseases (occurrence of new cases), mean age at onset of each disease, average duration of the disease, relative weight adjudicated to each disease with total health in its treated form and in its untreated form, proportion of the population with the disease who received treatment, structure of the population where the disease occurred, life expectancy of the population, and population's mean age at death (to obtain the last two inputs, the general mortality in the population was required), all the previous data were disaggregated by gender and age group 13.

This is how the information management consisted in collecting the mortality data for 2010 in the Department of Nariño based on official data, revised and validated for the country through vital statistics records of the Administrative Department for National Statistics (DANE, for the term in Spanish); this information was disaggregated by the mortality data for the 13 sub-regions: *Sanquianga, Pacífico Sur, Telembí, Pie de Monte Costero, Ex Provincia de Obando, Sábana, Guambuyaco, Abades, Occidente, Cordillera, Centro, Juanambú, *and* Río Mayo*, all through variables of age, gender, and code of basic cause of death according to the Global Burden of Disease (GBD) list. Also, morbidity data was collected for 2010 from the Department of Nariño based on official data, revised and validated for the country in the RIPS administrated by the Information Management System of the Ministry of Health and Social Protection (SISPRO), whose data cube was accessed from the Nariño Departmental Institute of Health (*Instituto Departamental de Salud de Nariño*, IDSN).

The inclusion criteria used were: people who fell ill or dies due to any of the causes consigned in the GBD according to vital statistics and the Individual Registry of Service Provision during the 2010 period; while the exclusion criteria were: people who were not registered in these sources. The following indicators were established as result variables: 1. YLPM: Years of life lost due to premature mortality; 2. YLLD: Years of life lost due to disability and, 3. DALYs: Years of life potentially lost, that is, the sum of years lost due to mortality and disability. The following exposure variables were established: 1. Gender: male and female; 2. Age groups: 0-4 years, 5-14 years, 15-44 years, 45-59 years, and 60 years and older; 3. Causes of Disease: Communicable, Non-communicable, and Externalities; 4. Incidence; 5. Mortality and, 6. Lethality. 

Consent to start the data collection process was approved by the IDSN Direction. The proposal was socialized with the members of the IDSN primary committee prior to beginning the collection, resolving doubts, and guaranteeing information on the presentation and publication of results to learn of the scope and limitations of the study. 

The analysis consisted in calculating the YLPM for which we proceeded to measure the years of life lost due to premature mortality (Duration x Number of deaths per cause); obtained through the GESMOR 14 software. 

• Direct calculation of YLPM with adjustments of life expectancy and reassignment of unspecific causes by sub-regions of the department.

• The study worked with disaggregated diseases or events that totaled 85% of all the YLPM for the calculation of DALYs.

To calculate disability-adjusted life years (DALY), weight of disability x duration x incidence were measured by using the weights for disability from a European study denominated "Dutch and Victorian burden of disease studies" 15 carried out by the creator of the DALYs. Lack of local records of incidence or prevalence is often solved using estimates for groups of countries or cities of similar socioeconomic and epidemiological patterns. The Nariño have a local cancer registry in the Municipality of Pasto, so for the incidence of cancer, the registry information of Gastric Cancer Pasto was used for the period 2003-2008. Weights due to disability were an important factor that reflected the severity of the diseases, given that they reflect the preferences or values attributed to the different states of health. In calculating DALYs, the mean population weight was used rather than that of the individual values. Weights were estimated by using people who were ill or had died due to different causes; this reflected the values attributed to the different states of health. These severity values ranged in a scale from 0 (full health) to 1 (a state of health equivalent to death). This phase was carried out with support from the DISMOD II program (open software by the WHO), which is a mathematical model developed by the WHO 16 to obtain consistent estimations of the incidence, prevalence, and mortality of a health problem within a specific population. 

Thereafter, through the GESMOR 14 software, the base tables were obtained to calculate DALYs for each disease per gender and age groups. Finally, the calculation of DALYs was obtained, which is the sum of YLPM and AVDP. For their understanding, rates and percentages of DALYs, YLPM, and YLLD were calculated for every 1,000 inhabitants, by age group and gender, and tables and graphics were also constructed.

It should be highlighted that the main biases that could arise were related to information, given that these types of studies depend on sources and information systems on mortality and morbidity, which habitually have systematic diagnostic bias, incorrect or incomplete death certificates, incorrect interpretation of the regulations of the International Classification of Diseases (ICD), and variations in the use of coding categories due to unknown or poorly defined causes.

The methodology proposed emerges from the need to understand the Department's health situation, in light of the existence of a substantial difference in income levels in the country, measured through Gross Domestic Product (GDP), for example, when comparing the wealthiest region: Bogotá, with one of the least favored: the Department of Nariño, the difference will be 3.7 times for 2010. Added to this, the Department has a coastline on the Pacific, which due to its geographic, economic, and social conditions presents the most negative indicators of unmet basic needs in the country, especially regarding undernourishment, quality and coverage of drinking water and basic health, and with respect to determinants like unemployment and family income, drug addiction, violence, and poverty in general. The Department has rural poverty rates that reach 59.3% and its municipalities can have poverty rates as high as 65, 80, and even 100%; while urban poverty reaches a Departmental average of 26.1%. Unlike the national population pyramid, that of Nariño is wide at the base, which demonstrates the existence of a large portion of young population. All this indicates that the demographic transition process has been taking place more slowly in the Department than in the country, but with a tendency being equated in the following years, due to tendencies for declined fertility and general mortality, as well the urbanization process being undertaken in the Department, except for some zones like the Pacific zone 17.

## Results

**Figure 1.**
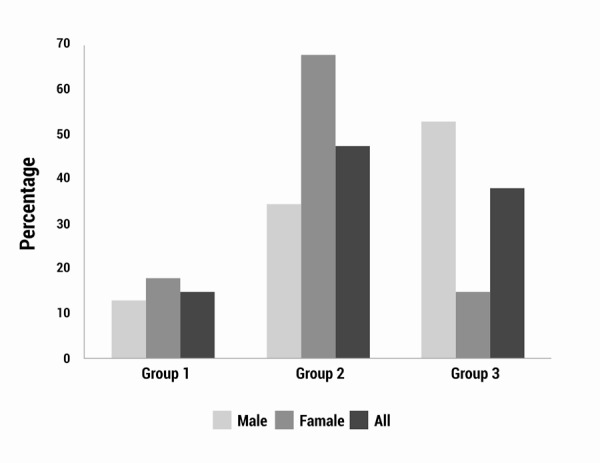
Percentage distribution of DALYs by disease groups according to sex in the Department of Nariño, 2010.

The burden of disease in the Department of Nariño was estimated in 2010 at 88,423 total DALYs and a DALY rate of 53.9 per every 1,000 inhabitants. The group of diseases with more DALYs corresponds to non-communicable diseases, with 47%, that is, 41,855 DALYs and a rate of 25.5 per every 1,000 inhabitants. According to gender in the Department, women have 68% DALYs or 23,273 DALYs of non-communicable diseases; while men have 35% of the DALYs or 28,258 DALYs of accidents and lesions. In the group of communicable, maternal, perinatal, and nutritional diseases there is similarity between men and women; men have 6,932 DALYs and a rate of 8.4, while women have 6,147 DALYs and a rate of 7.5. The prevalence of DALYs in the Department of Nariño according to age group between 0 and 4 years for the group of communicable, maternal, perinatal, and nutritional diseases with 6,513 DALYs or 55.6%; in the group representing those 60 and more years due to non-communicable diseases with 14,342 DALYs or 87.5%; and in the group 15 to 44 years due to accidents and lesions with 26,991 DALYs or 64.5% (Table 1 and Fig. 1). 

**Table 1.**
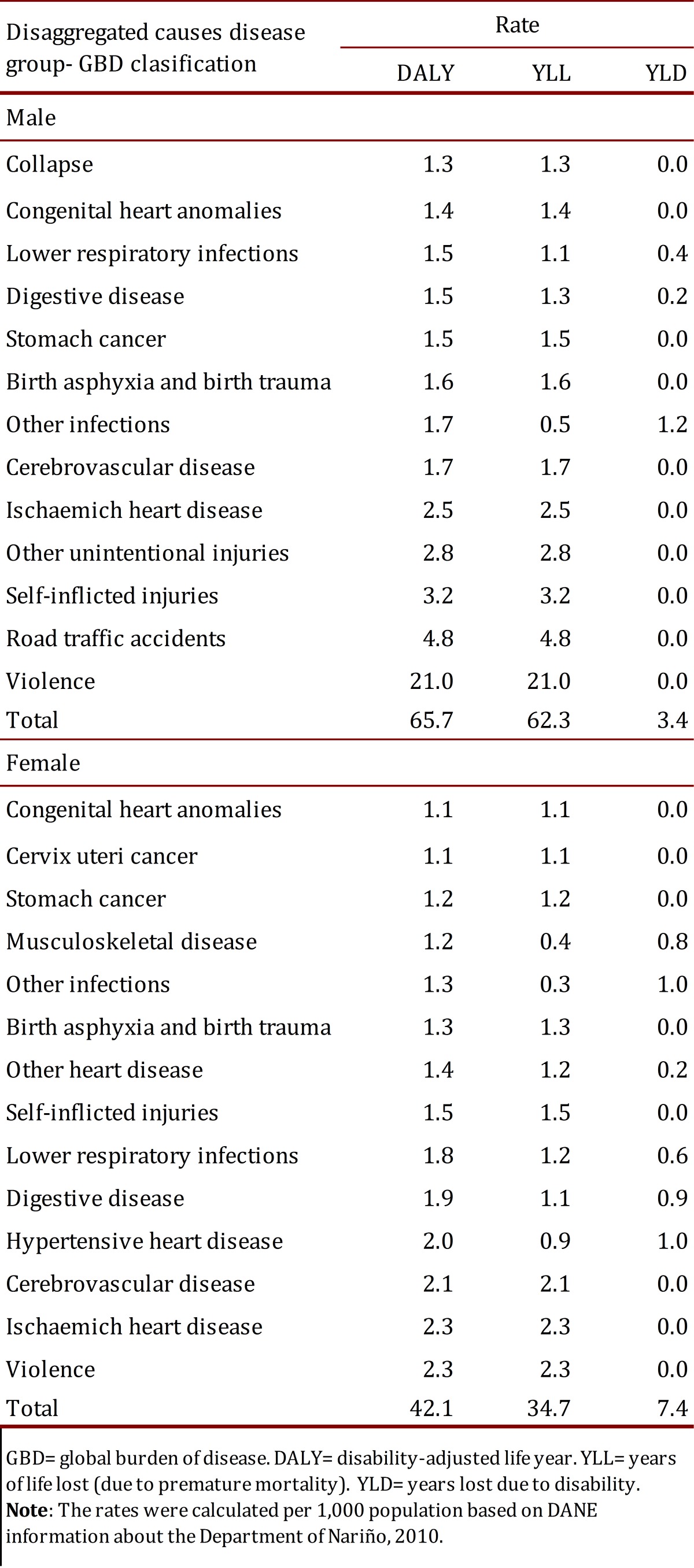
Leading causes of disease in male and female according YLL and YLD in the Department of Nariño, 2010.

The YLPM or DALYs due to mortality notably surpassed YLLD or DALYs due to disability in both men and women. In men, DALYs due to mortality represent 94.9% and 82.5% in women. Although the highest concentration of YLLD is found in the age group from 5 to 14 years in both genders, it should be highlighted that in men it represents 37.0% of DALYs, but in women it represents 59.9%. The YLLD of women were concentrated in non-communicable diseases with 4,515 or 75.0%; while in men, YLLD were concentrated in the group of communicable diseases with 1,435 or 51.9%. The YLPM in women were also concentrated in the group of non-communicable diseases with 18,758 or 66.1% of the YLPM; while in men, the YLPM were concentrated in the group of accidents and lesions with 28,506 or 55.6% of the YLPM (Table 1 and Fig. 1). 

It must be highlighted that the first cause of disease in women from the Department of Nariño corresponds to violence with 1,914 DALYs, a DALY rate of 2.3, YLPM of 1,910, and YLLD of 5; the following four causes correspond to non-communicable diseases, like ischemic cardiovascular disease with 1,895 DALYs, a DALY rate of 2.3, YLPM of 1,881, and YLLD of 14; cardio-cerebrovascular disease with 1,713 DALYs, a DALY rate of 2.1, YLPM of 1,705, and YLLD of 8; hypertensive cardiovascular disease with 1,596 DALYs, a DALY rate of 2.0, YLPM of 749, and YLLD above the other causes with 848 and other diseases of the digestive system with 1,593 DALYs, a DALY rate of 1.9, YLPM of 883 and YLLD of 711. The following nine causes of disease correspond to communicable diseases like lower respiratory tract infections with 1,445 DALYs, a DALY rate of 1.8, YLPM of 985, and YLLD of 460; anoxia, asphyxia, and trauma at birth with 1,058 DALYs, a DALY rate of 1.3, YLPM of 1,054, and YLLD of 3; other infections with 1,051 DALYs, a DALY rate of 1.3, YLPM of 274, and YLLD of 776; other cardiovascular and osteomuscular diseases, congenital anomalies, malignant tumors, and cervical tumors with 99.0% YLPM. The first four causes of disease in men from the Department of Nariño correspond to accidents and lesions, specifically to violence with 17,286 DALYS, a DALY rate of 21 per every 1,000 inhabitants, YLPM of 17,285, and YLLD of 1; circulation or traffic-related accidents with 3,974 DALYs, a DALY rate of 4.8, YLPM of 3,974, and YLLD of 1; suicide with 2,650 DALYs, a DALY rate of 3.2, YLPM of 2,648, and YLLD of 1; and other accidents with 2,338 DALYs, a DALY rate of 3.2, YLPM of 2,321, and YLLD of 17. The following two causes correspond to non-communicable diseases like ischemic cardiovascular disease with 2,024 DALYs, a DALY rate of 2.5, YLPM of 2,018, and YLLD of 7; and cardio-cerebrovascular disease with 1,398 DALYs and a DALY rate of 1.7, YLPM of 1,392, and YLLD of 6. The other eight causes correspond to communicable diseases, specifically other infections, which is the cause with more YLLD in men with 1,022; anoxia, asphyxia, trauma at birth and lower respiratory tract infections with 1,199 DALYs; also highlighted are malignant stomach tumor, congenital cardiac anomalies and other diseases of the digestive system; while accidents and lesions highlight falls and drowning (Table 1 and 2 ).

**Table 2.**
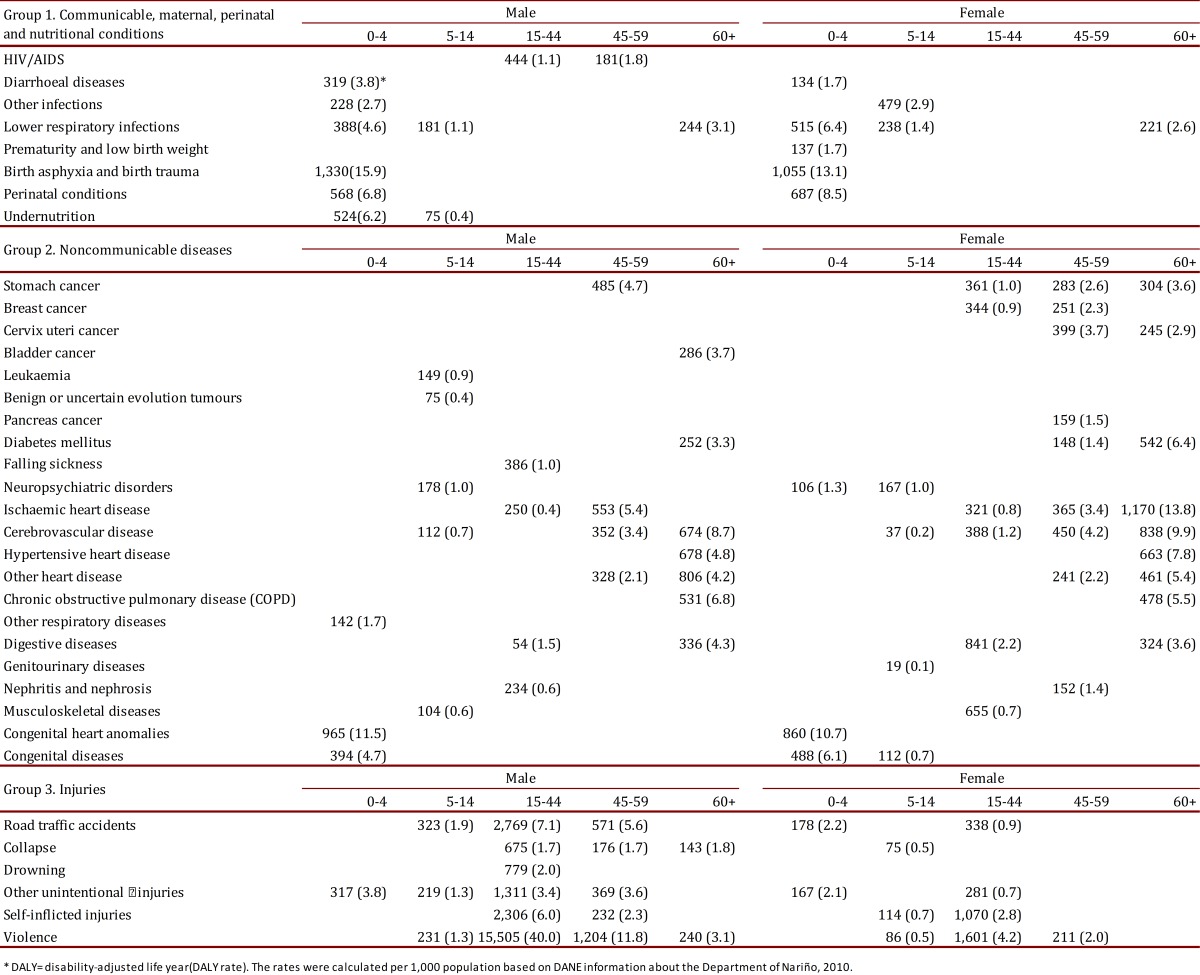
Summary of DALYs and DALY rate of the ten leading causes of disease by sex and age group in the Department of Nariño, 2010.

It should be mentioned that the distribution of causes of disease in children behaves differently from that for adults. According to the age group, among the first 10 causes of disease in males in the age group from 0 to 4 years, six correspond to communicable, maternal, perinatal, and nutritional diseases, like: diarrhea, other infections, lower respiratory tract infections, other perinatal diseases, protein malnutrition, highlighting anoxia, asphyxia, trauma at birth as first cause with 1,330 DALYs and a DALY rate of 15.9. Also, three causes correspond to non-communicable diseases, like: other respiratory diseases, other congenital diseases, and congenital cardiac anomalies. Another cause corresponds to accidents and lesions as other accidents. Among the first 10 causes of disease in women, five correspond to communicable diseases like: diarrhea and low weight/prematurity, lower respiratory tract infections, other perinatal diseases, with anoxia, asphyxia, trauma at birth highlighted in males with 1,055 DALYs and a DALY rate of 13.1. Three causes correspond to non-communicable diseases, among them neuropsychiatric diseases, other congenital diseases, with congenital cardiac anomalies highlighted in males with 860 DALYs and a DALY rate of 10.7. Another two causes in girls correspond to circulation accidents and other accidents. Among the first 10 causes in men in the age group 5-14 years, two causes correspond to the group of communicable, maternal, perinatal, and nutritional diseases: lower respiratory infections and protein-caloric malnutrition. Five causes correspond to non-communicable diseases like leukemia, other neuropsychiatric diseases, cardio-cerebrovascular disease and other osteomuscular diseases; likewise, three causes correspond to accidents and lesions like: circulation accidents, other accidents and violence. Among the first 10 causes of DALYs in women from the age group 5-14 years, two correspond to communicable diseases as other infections and lower respiratory tract infections; four causes of diseases correspond to non-communicable diseases like: other neuropsychiatric diseases, cardio-cerebrovascular disease, other genitourinary diseases, and other congenital diseases (Table 2).

It is worth mentioning that in the distribution of causes of disease in adults, non-communicable diseases and accidents and lesions prevail. Among the first 10 causes of disease in men from the age group 15-44 years, the causes of disease and their DALYs are concentrated on accidents and lesions, where violence accumulates a large part of the DALYs with 15,505 and a DALY rate of 40.0, and consecutively circulation accidents, suicide, other accidents, and falls. Four of the causes of disease correspond to non-communicable diseases like: epilepsy, ischemic cardiovascular disease, nephritis/nephrosis and diseases of the digestive system. A very important cause of communicable diseases is AIDS with 444 DALYs and a DALY rate of 1.1. Causes of disease in women in the age group 15-44 years are, similarly, concentrated in groups 2 and 3. In the first we find malignant stomach tumor, malignant breast tumor, ischemic cardiovascular disease, cardio-cerebrovascular disease, diseases of the digestive system, and osteomuscular diseases; while the non-communicable group highlights violence, suicide, circulation accidents, and other accidents. Among the first 10 causes of disease in men in the age group 45-59 years, we again find violence, circulation accidents, other accidents, suicide, and falls. Among the non-communicable diseases, there are ischemic cardiovascular disease, malignant stomach tumor, cardio-cerebrovascular disease, and other cardiovascular diseases; the group of communicable diseases again highlights AIDS with 181 DALYs and a rate of 1.8. Among the first 10 causes of disease in women in the age group 45-59 years, non-communicable diseases prevail like cervical malignancy, malignant stomach tumor, malignant breast tumor, ischemic cardiovascular disease, cardio-cerebrovascular disease, other cardiovascular diseases, pancreatic malignant tumor, nephritis/nephrosis, and diabetes mellitus; group 2 highlights violence. Among the top 10 causes of disease in men in the age group 60 years and older, non-communicable diseases are highlighted like: other cardiovascular diseases, hypertensive cardiovascular disease, cardio-cerebrovascular disease, chronic obstructive pulmonary disease (COPD), other diseases of the digestive system, diabetes mellitus, and malignant prostate tumor; among accidents and lesions, violence and falls prevail; and the group of communicable diseases spotlights lower respiratory tract infections. Among the top 10 causes of disease in women in the age group 60 years and older, no accidents and lesions are present but ischemic cardiovascular disease is spotlighted, along with cardio-cerebrovascular disease, hypertensive cardiovascular disease, chronic obstructive pulmonary disease, other cardiovascular diseases, other diseases of the digestive system, diabetes mellitus, cervical malignancy, and malignant stomach tumor. Group 1 highlights lower respiratory tract infections (Table 2).

## Discussion

Direct measurement of health as a positive concept is difficult, which is why currently complex measures are used to describe and summarize the epidemiological behavior of disease or, in other words, the measurable deviation of the state of health. Among the gap estimators that measure the magnitude of the difference in years of healthy life existing between a population's real state of health and an ideal state as object of this study, it is supported on the calculation and interpretation of DALYs. This complex indicator proposes a health-disease state different from complete and total health, given two consequences of disease: mortality and disability. A big limitation of DALYs is that its starting point is a model situation where all the members of the population within the same age and gender have the same life expectancy, hence, every premature mortality is defined from the diseases or lesions occurring prior to arriving at said expectation; thereby, in that population in general terms the difference between real age at death and life expectancy is considered lost time; all this with the sole purpose of introducing a comparison factor 18.

Although the results in this study were close to the WHO's global prognosis that estimates that for 2020 non-communicable diseases will account for two thirds of the global pathological burden 19 as long as Colombia propitiates a future scenario of reforms, political and social transformations, of reconciliation and peace; it is difficult to ensure this for the Department of Nariño, given that in addition to the increase of the burden of this group of diseases, accidents and lesions also concentrate much of the DALYs from the Department. This is a phenomenon with greater occurrence in men in economically active ages and due to homicides, suicides, and traffic accidents, and like low-income countries the Department has the highest burden of disease due to communicable diseases. It is evident that this burden of disease can be determined by peculiarities of the region and the country, which endure the increasing attacks of the armed conflict, organized delinquency, intrafamily violence and violence against women, the economic crisis and restitution of lands ravaged by drug trade and war, rampant intolerance in the streets and roadways of urban zones 20, just to mention some of the problems faced by the country and the region. 

The results of this study differ from the WHO report on Latin America and the Caribbean, whose burden of disease is concentrated on non-communicable diseases. The aforementioned is supported on the prevalence of DALYs of communicable diseases in the Department. These results also differ with respect to the first causes, given that in the Americas and the Caribbean neuropsychiatric conditions, ischemic heart disease, cerebrovascular disease, and diabetes 9 prevail, but the Department of Nariño highlights violence, suicide, all cardiovascular diseases, malignant tumors, and infections in children, hence, many questions emerge about the health system in Colombia, which is being reformed amid multiple social mobilizations. These questions focus on two potential situations: the first, will the national government provide the assistance required in confronting the Department's problems in the long term, and - secondly - will said assistance reduce the impact of non-communicable diseases and lesions amid programs implemented in multi-sector manner from primary and secondary prevention. The prior concern is added to a global panorama, where 90% of the global healthcare expense is invested by developed nations that only have 20% of the world's population, and to the priority investment of public health expense in causes of disease and death like infections, infant malnutrition, diabetes, obesity, cancer, and cardiovascular diseases 21; and contradictorily to neglect of violence.

In 1990, throughout the world, communicable diseases caused 59% of the deaths and disability among the poorest countries, while in the wealthiest countries non-communicable diseases caused 85% of the deaths and disability. A decrease in communicable diseases between 1990 and 2020 increased life expectancy among the poorest in the world 18. Nevertheless, within Colombia this panorama has its own peculiarities, for example, if we contrast the rates of communicable and non-communicable diseases during 2005 - according to the latest study on burden of disease in Colombia per departments - we may find that on average non-communicable diseases were more frequent than communicable diseases. However, upon analyzing morbidity between 2004 and 2008, it is shown that in the Department of Nariño, as well as in Atlántico, Bolívar, Huila, Norte de Santander, and Putumayo communicable pathologies increase 22. The aforementioned shows that although the pathological burdens from non-communicable diseases and lesions, these should be of concern given their high contribution to mortality and disability in the department; communicable diseases are also of concern given their increase in recent years, thereby, Colombia has yet to have a complete epidemiological transition, given that diagnoses of non-communicable diseases and infections continue having relevant and different presence in all age groups and offer of health services in the regions.

This study has demonstrated that communicable diseases are more frequent in younger age groups, while non-communicable diseases appear mostly in older age groups. Externalities (accidents, self-inflicted lesions, and violence) affect more often groups between 5 and 44 years of age. These findings are important to rethink at the national level the development of health policies that prevent and protect the different population groups and overcome gaps in health services, especially second-level (tier two) care due to the absence of hospitals in the most remote zones. The most important finding in this study is that undoubtedly the group of causes of accidents and lesions cannot be overlooked in Nariño, especially regarding inflicted and self-inflicted violence, given that these are profound generators of burden of disease in the department. Besides problems with the health system, the Department has profound social, economic, and political problems, with the presence of armed groups being of greatest concern; all these contribute to the barriers of access to health services because they co-opt public funds for healthcare and keep people from mobilizing to urban zones with better access. Similarly, war is producing irreversible effects on women, indigenous groups, Afro-descendent groups, youth, and children, as well as on male fighters and families outside the conflict, exposing their right to constant vulnerability by forcing their displacement, confinement, and recruitment that disables and kills 23. 
